# Addressing antiretroviral therapy-related diagnostic coverage gaps across South Africa using a programmatic approach

**DOI:** 10.4102/ajlm.v7i1.681

**Published:** 2018-11-12

**Authors:** Naseem Cassim, Lindi M. Coetzee, Wendy S. Stevens, Deborah K. Glencross

**Affiliations:** 1 Department of Molecular Medicine and Haematology, University of the Witwatersrand, Johannesburg, South Africa; 2 National Priority Programme, National Health Laboratory Service, Johannesburg, South Africa

## Abstract

**Background:**

A major challenge facing South Africa is the concomitant HIV and tuberculosis epidemics. The National Health Laboratory Service provides testing for staging HIV-positive patients, monitoring patients on antiretroviral therapy (ART) and diagnosing tuberculosis. Not all health districts have equivalent ART-related coverage in particular for CD4 and HIV viral load testing.

**Objectives:**

The Integrated Tiered Service Delivery Model coverage precinct approach was used to address ART-related testing service coverage gaps in a manner that balances cost, quality and equity.

**Methods:**

An algorithm was developed to identify and address ART-related diagnostic coverage gaps. Data was extracted from the corporate data warehouse and Oracle systems for the period of April 2015 to March 2016. Daily test volumes were based on 21.73 working days per month. Data were analysed using MS Excel and mapped using ArcCatalog and ArcMap. Capacity analysis was informed by the available testing-platforms.

**Results:**

Health district daily HIV viral load volumes ranged from 2 to 1308 samples. Nineteen candidate laboratories were identified to address the coverage gaps. Following the proximity analysis, testing was consolidated at four candidate laboratories, resulting in 13 revised candidate laboratories. The revised candidate laboratory daily HIV viral load referrals ranged between 5 and 205 samples, with CD4 volumes between 6 and 85 samples. Remaining coverage gaps were identified in seven municipalities.

**Conclusions:**

The study demonstrated that the service coverage precinct approach could be used to identify coverage gaps for a defined ART-related testing repertoire.

## Introduction

A major challenge facing South Africa is the concomitant HIV and tuberculosis epidemics.^1,2^ It is estimated that 36.7 million people globally were living with HIV in 2016, with a prevalence of 0.8%, of which 25.5 million people live in sub-Saharan Africa (~70% of the global HIV burden).^3,4^ In South Africa, it is estimated that seven million people are living with HIV, with an adult prevalence rate of 19.2% in 2015.^4,5^ Despite many obstacles faced by South Africa between 2010 and 2014, the response to the AIDS epidemic resulted in the largest antiretroviral treatment (ART) programme in the world, with over two million HIV-infected people receiving treatment by 2010.^6^

In 2006, the South African government approved the ambitious National Strategic Plan for HIV and AIDS and sexually-transmitted infections (2007–2011) and committed the government to providing ART to 80% of those eligible.^7,8,9^ In 2010, with ~900 000 people on ART, South Africa launched the national HIV counselling and testing campaign that aimed to test 20 million people over 20 months.^7,8^

The required ART scale up in South Africa necessary to meet the HIV counselling and testing campaign targets led to the announcement that accreditation would be abandoned and that all public healthcare facilities would be geared up to provide ART.^7,8^ With the accreditation requirement removed, ART services were decentralised to the majority of health facilities over the next few years (*n* = ~3000).^7^ This change removed the need for a national team to accredit health facilities for ART provision. District coordinators could use a checklist to speed up the expansion of ART services to the community level. The introduction of Nurse Initiated Management of Antiretroviral Treatment at primary health care facilities facilitated further decentralisation of ART, strengthened retention of patients in care and reduced the burden of managing uncomplicated cases at referral hospitals.^10,11^

Currently, the Joint United Nations Programme on HIV/AIDS (UNAIDS) estimated that seven million people are living with HIV in South Africa with a prevalence rate of 19.2% for adults (>15 years).^12^ UNAIDS also reported 380 000 new HIV infections in 2015, with 180 000 deaths due to AIDS.^12^ By 2015, 3.3 million individuals were on ART, resulting in 48% coverage of all HIV-positive individuals.^5^

In 2015, six countries accounted for 60% of the global tuberculosis burden, with the highest burden in India, followed by Indonesia, China, Nigeria, Pakistan and South Africa.^13,14^ Tuberculosis is also a leading killer of HIV-positive people (35% of deaths were due to tuberculosis).^13^ By 2015, a tuberculosis incidence of 834 cases per 100 000 population was reported for South Africa (includes HIV-positive tuberculosis cases).^13^ The incidence of multidrug-resistant tuberculosis was 37 cases per 100 000 population.^13^ Tuberculosis treatment coverage was estimated to be 64% overall, increasing to 97% for patients with a known HIV status.^13^ Eighty-five per cent of patients in HIV/ tuberculosis care were on ART. South Africa developed the National Tuberculosis Programme that aimed to find, treat and prevent tuberculosis in order to avoid tuberculosis deaths and reduce transmission.^14^ The National Tuberculosis Programme has substantially strengthened the national tuberculosis control programme.^14,15^ Significant milestones for the National Tuberculosis Programme include the implementation of directly-observed treatment short course,^15^ introduction of fixed-dose combination drugs, conducting the national drug-resistance survey, introduction of Hain MTBDRplus (multidrug-resistant tuberculosis rapid test) and Xpert MTB/RIF (replacement for sputum smear microscopy).^14^ Significant challenges, however, remain to reduce the tuberculosis burden in South Africa.

The laboratory service plays a critical role in diagnosing tuberculosis and monitoring treatment.^16,17^ Similarly, the laboratory service is required to evaluate patients and monitor virological suppression once on ART.^18^ In South Africa, the National Health Laboratory Service (NHLS) provides access to diagnostics to 80% of the population attending public sector health care facilities through a network of laboratories across nine provinces.^19^ The NHLS aims to provide quality, affordable and sustainable health laboratory medicine, provide training for health science education and undertake innovative and relevant research.^19^ Routine clinical pathology services are offered at all 288 laboratories providing routine haematology, chemical pathology and microbiology testing.

For the HIV programme, the NHLS provides CD4, HIV viral load, Xpert MTB/RIF and other routine testing to support ART initiation, as defined in the national guidelines.^18^ CD4 testing within the NHLS is standardised using flow cytometers (Beckman Coulter, Miami, Florida, United States) and the PanLeucoGating method.^20,21^ CD4 laboratories use either the MPL/CellMek platform at busier laboratories or the Aquois system for lower throughput laboratories.^22,23,24^ There are currently 49 laboratories offering CD4 testing. HIV viral load testing is offered using the Roche (Roche Diagnostics, Basel, Switzerland) and Abbott (Abbott Molecular, Abbott Park, Illinois, United States) platforms. Laboratories allocated to the Roche Diagnostic platform use the HIV-1 Test (v2.0) assay on either the COBAS AmpliPrep/COBAS TaqMan, COBAS 6800 or COBAS 8800 analysers.^25^ Additionally, some laboratories allocated to the Abbott Molecular platform use the RealTi*m*e HIV-1 assay with the Abbott *m*2000 RealTi*m*e System (m2000sp and m2000rt) analyser.^26^ HIV viral load testing is currently offered at 17 laboratories using both the Roche and Abbott assays. Xpert MTB/RIF testing is based on the Cepheid (Sunnyvale, California, United States) GeneXpert platform.^27,28^ The GeneXpert technology is a modular system and supplied as a GX4 (i.e., four modules), GX16, GX48 and GX80.^29^ There are 214 NHLS laboratories offering Xpert MTB/RIF testing, using a decentralised approach across all 52 health districts.^30^ However, not all health districts have equivalent coverage for CD4 and HIV viral load testing.

The Integrated Tiered Service Delivery Model (ITSDM) described for CD4 testing across South Africa to achieve coverage, proposed 104 CD4 testing sites consisting of 60 laboratories (Tier-3 to Tier-5), 22 point-of-care (POC) hubs (Tier-2), and 22 decentralised POC testing sites (Tier-1) that would only service one health facility.^31^ In particular, tiers two and three were identified as potential operational platforms to enable extending HIV testing services to existing NHLS laboratories. The aim of the latter was to deliver equitable access to laboratory services by providing technology in the respectively identified laboratory that appropriately matched service delivery requirements within the defined service coverage precinct of the laboratory in question.^31,32^ The efforts to date have been test-specific (CD4-linked) and as such, a new analysis that optimally addresses ART-related testing coverage gaps is needed. For example, adding only HIV viral load testing to a rural district laboratory repertoire is not sufficient to meet the needs for basic ART-related test repertoire. To improve diagnostic coverage, repertoire testing demands include concurrent access to CD4, cryptococcal antigen (CrAg), Xpert MTB/RIF, creatinine (including estimated calculation of glomerular filtration rate), alanine transaminase (ALT), full blood count (FBC), and Hepatitis B surface antigen tests.^18^

A national burden of disease study conducted between 1997 and 2010 by the South African Medical Research Council reported that the four major broad causes of death in South Africa were HIV and tuberculosis, non-communicable diseases, injuries, and other Type One conditions such as nutritional deficiencies.^33,34,35^ The burden of these non-communicable diseases will probably increase as the roll-out of ART scales up further and reduces mortality from HIV.^34^ The scale of the challenge posed by the combined and growing burden of HIV, tuberculosis and non-communicable diseases demands an extraordinary response from the health care system.^34^

The lessons learnt from implementation of the CD4 ITSDM^31^ and other related work^36,37^ to address coverage will be used to investigate coverage gaps for a defined ART-related test repertoire. The aim of this study is to use the existing NHLS laboratory capacity to address ART-related coverage gaps and deliver services where some testing gaps exist in a sustainable manner that balances cost, quality and coverage.

## Methods

### Ethical considerations

Ethics clearance for this work was obtained from the University of the Witwatersrand (study number: M1706108). This study involved the secondary analysis of laboratory test volumes data that does not contain any patient identifiers. No patient recruitment was necessary as routine laboratory data was used for the study.

### Study design

**FIGURE 1:**
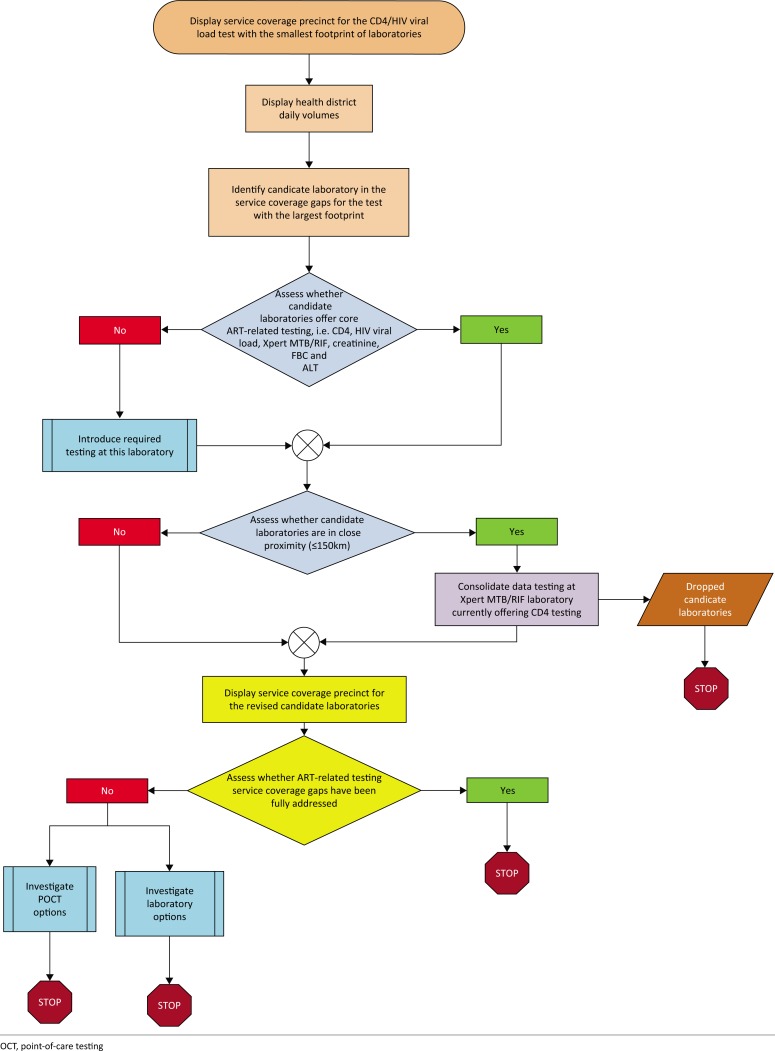
Flow diagram steps used to identify candidate laboratories to address antiretroviral therapy-related testing coverage gaps, South Africa.

This study is based on the lessons learnt from the ITSDM and extends these concepts to develop an algorithm that addresses service coverage for a defined test repertoire. The algorithm developed was used to identify existing NHLS laboratories (candidates) that are not currently offering HIV viral load and CD4 testing that could be used to offer these diagnostic services and address ART-related testing coverage gaps (Figure 1). Laboratories were assigned an alias in each province, e.g. EC1 for the first laboratory alphabetically in the Eastern Cape province. As the HIV viral load test currently has the smallest national service footprint, this test will be used as the baseline test from which to assess coverage gaps.

The seven steps used in the algorithm are described below:

### Step 1: Display service coverage precinct for the HIV viral load test

A list of HIV viral load laboratories was compiled, including latitude and longitude coordinates. These data were collected using a spreadsheet and converted to a shapefile using ArcCatalog (Redlands, California, United States) and loaded as a new layer on ArcMap (Redlands, California, United States)^38^ (displayed as a blue circle). The analysis toolbox was used to access the proximity toolset to display a 250 km buffer (equivalent to 2.5–3 h travel time depending on road infrastructure) around each HIV viral load laboratory to represent the current service coverage precinct. Health facilities within the current HIV viral load service coverage precinct were assumed to have adequate access to testing.^31^ Areas outside the service coverage precinct were assumed to lack coverage, requiring additional testing sites to address gaps.

### Step 2: Display health district daily volumes

HIV viral load samples tested within the NHLS are recorded in the laboratory information system through a data interface to the analyser. Specimen-level laboratory information system data are then replicated to the corporate data warehouse for national programmatic reporting. Health district HIV viral load test volumes for the period April 2015 to March 2016 (fiscal year [FY]2015/2016) were extracted from the corporate data warehouse. The data extract included the health district name and total HIV viral load test volumes, that is, the number of samples tested in the FY2015/2016. Daily health district HIV viral load test volumes were calculated assuming an average of 21.73 working days per month and 12 months per year. In South Africa, the Municipal Demarcation Board is responsible for determining municipal boundaries.^39^ It also provides spatial data files at the provincial, district, municipal and ward levels.^39^ Health district shape files were downloaded from the Municipal Demarcation Board website^39^ and edited using ArcCatalog to add a new data field to capture district daily HIV viral load volumes (integer data type).^38^ The shapefile was added as a new layer on ArcMap^38^ and edited to manually capture daily HIV viral load test volumes for 52 health districts.

Using the symbology functionality in ArcMap,^38^ the daily HIV viral load volumes were displayed using quantiles with graduated colours (across six classes) for easy visual interpretation. Each class was allocated a different colour, namely: 8–16, dark green; 17–64, green; 65–150, light green; 151–300, orange; 301–600, light red; and 601–1380, dark red. The cut-off values were adjusted manually for the first two classes to reflect the daily capacity of the GeneXpert GX4 (16 samples per day) and the GX16 (64 samples per day).

### Step 3: Identify a candidate laboratory in the HIV viral load service coverage gap areas

A Microsoft Excel list of NHLS laboratories that do not currently provide HIV viral load testing, with their respective latitude and longitude coordinates, were loaded as a new layer on ArcMap. The maps were printed on an A3 paper in colour and given to three individuals to identify candidate laboratories in the current HIV viral load coverage gaps. The three lists were reviewed to generate the approved list of candidate laboratories. This list was converted to a shapefile using ArcCatalog.^38^ This shapefile was then added as a new layer of the map and visualised as red circles using the symbology function in ArcMap.^38^

### Step 4: Assess whether candidate laboratories offer antiretroviral therapy-related repertoire testing

For each test performed within the NHLS network, the laboratory information system generates single or multiple tariff code or codes to generate expenditure data (e.g. tariff code 3020 is allocated to the glucose test). These data are used to generate accounts on the Oracle enterprise resource planning system in the accounts receivable module,^40^ which generated the volume and revenue report that details test volumes by tariff code. The tariff code volumes provide an easy analysis of test volumes where a one-to-one relationship exists between a test and its respective tariff code. This is the case for the ART-related test repertoire. Year-to-date volumes and revenue data from the March report, namely FY2015/16 volumes,^41^ were used. The report includes cost centre numbers, laboratory name, tariff code, tariff description, year-to-date test volumes and referred test volumes. Referred test volumes indicate the number of tests referred to another laboratory when this test is not offered by the receiving laboratory. The data extract was analysed using Microsoft Excel to determine: (1) financial year 2015/16 CD4, creatinine, ALT, FBC and Xpert MTB/RIF test volumes for each laboratory; and (2) where these tests are not offered, the referral test volumes. The aim of this analysis was to identify whether the candidate laboratories currently provide the required test repertoire. Tariff codes were identified for the HIV viral load, CD4, CrAg, ALT, FBC and Xpert MTB/RIF tests to assess year-to-date testing and referred volumes. The testing status of each candidate laboratory was to be reported as a table, meaning that if no test volumes were found for a specific test (tariff code) in FY2015/16, it was assumed the laboratory did not offer testing.

### Step 5: Assess candidate laboratories in close proximity (≤ 150 km) to avoid over-capacitation

In a geographic area within a 150 km circumferential service coverage precinct, one laboratory would be able to provide sufficient coverage, removing the need to over-capacitate multiple laboratories. The aim of this step was to prevent over-capacitation and provide optimal testing at a candidate laboratory with the required test repertoire. An independent visual inspection was again conducted by three single individuals. They were requested to identify laboratory clusters on the printed map for candidate laboratories in close proximity. The responses were consolidated to identify clusters. For each cluster identified, Google Maps was used to determine inter-laboratory distances (kilometres) and drive times (minutes). The Google Maps drive time is not able to factor aspects such as stop or go sections due to road construction, adverse weather, traffic congestion and terrain and road condition. Where laboratories in a cluster were >150 km from each other, they were added to the list of revised candidate laboratories, as the laboratories were too far apart for consolidated testing. Where the inter-laboratory distance was ≤ 150 km for laboratories in a cluster, the laboratory currently offering CD4 testing and reporting the highest test volumes was the preferred choice for consolidation in the cluster. Where consolidation was indicated, the combined HIV viral load and CD4 volumes were used to determine the required testing capacity.

Where a candidate laboratory did not currently offer HIV viral load or CD4 testing based on volumes and revenue year-to-date data, referred test volumes were used. All daily volume calculations assumed 21.73 working days per month and 12 months per year. This assumption is based on small district (rural) laboratories that offer an 8-hour service, but may not be applicable to larger laboratories that offer a 24-hour service impacting on testing capacity.

The capacity analysis was done in consultation with three individuals from the National Priority Programme unit, with expertise in either CD4 or HIV viral load testing. One HIV viral load and CD4 platform was then allocated to each candidate laboratory based on the daily volumes or referrals (consolidated volumes where applicable). For the purpose of this paper, daily CD4 volumes of less than ten samples were allocated to the FacsPresto (Becton Dickinson, San Diego, California, United States) platform.

### Step 6: Display service coverage precinct for the revised candidate laboratories

In the identified clusters where consolidation was proposed, excluded laboratories were removed to generate a list of revised candidate laboratories. The list of revised candidate laboratories, with latitude and longitude values, was converted to a shapefile using ArcCatalog and added as a new layer (reported as red circles) on ArcMap.^38^ A service coverage precinct buffer (250 km) was drawn around the laboratory on ArcMap using the proximity tool to assess the additional service coverage the precinct provided (yellow circle).^38^

### Step 7: Assess whether antiretroviral therapy-related testing service coverage gaps have been fully addressed

For municipal areas without adequate coverage, town names were added to ArcMap. For these areas, either POC testing or additional laboratory sites are proposed.

## Results

### Daily district HIV viral load test volumes

**FIGURE 2:**
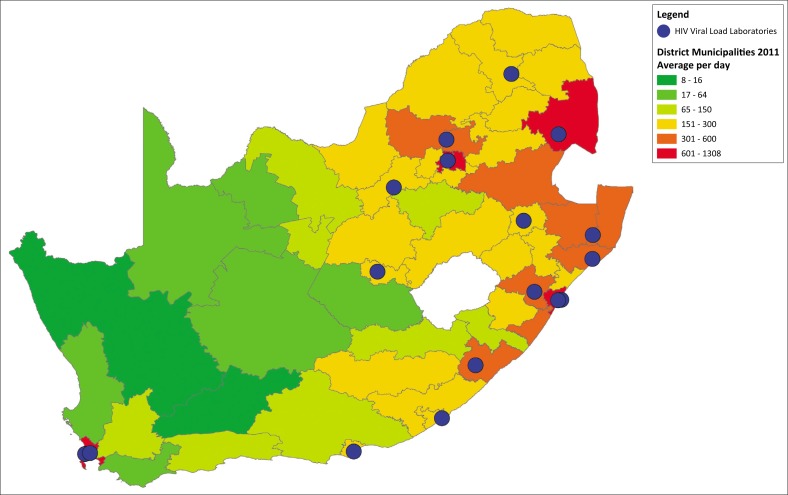
Current daily HIV viral load test volumes per day per health district with the laboratories offering testing (n=17), South Africa.

Only 5 out of 52 districts reported daily HIV viral load test volumes between 601 and 1308, namely City of Cape Town Metro, City of Johannesburg Metro, Ehlanzeni, Ekurhuleni Metro and eThekwini Metro (Figure 2). These health districts requested 33% of the national HIV viral load test volumes.

Nine health districts reported daily HIV viral load test volumes between 301 and 600; 22 health districts reported a daily HIV viral load test volume between 151 and 300 samples; only eight health districts had daily HIV viral load volume between 1515 and 300 while just 6 districts reported between 16 and 64 tests per day.

There were two districts that reported daily volumes ≤ 16 samples per day, namely Central Karoo and Namakwa health districts.

### Candidate laboratories in the HIV viral load service coverage gaps

**FIGURE 3:**
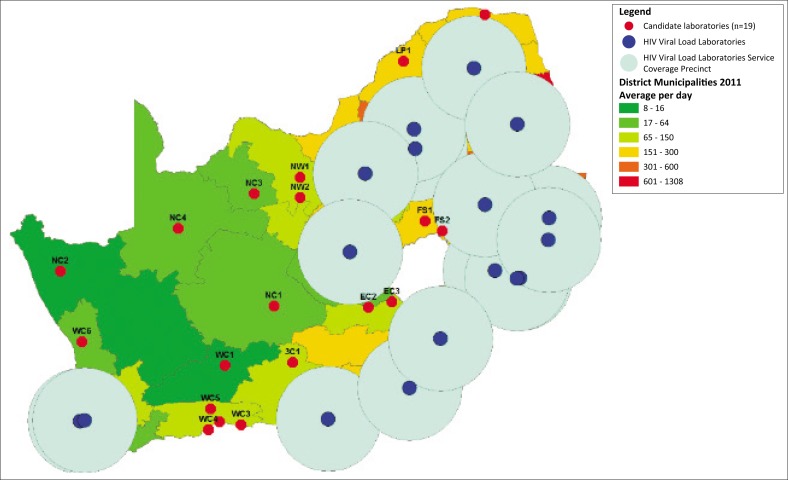
Current HIV viral load coverage provided using a service coverage precinct of 150 km circumference and the identification of candidate laboratories in the coverage gaps (*n* = 19), South Africa.

The ArcMap analysis reported adequate HIV viral load coverage in the Gauteng, KwaZulu-Natal and Mpumalanga provinces (Figure 3). Poor coverage was identified across all districts in the Northern Cape province. Selected health districts with poor coverage in the Western Cape, Eastern Cape, North West, Free State and Limpopo provinces were also identified. Nineteen candidate NHLS laboratories were identified to scale up services (Table 1). Of these candidate laboratories, six were identified in the Western Cape province (WC1–WC6). Four candidate laboratories were identified in the Northern Cape province (NC1–NC4) and three in the Eastern Cape province (EC1–EC3). Only two candidate laboratories were identified in each of the Limpopo (LP1–LP2), Free State (FS1–FS2) and North West (NW1–NW2) provinces.

**TABLE 1:** Current testing offered by the candidate laboratories to address the antiretroviral therapy-related testing coverage gaps, South Africa.

Number	Province	Laboratory alias	Testing offered on-site							

			HIV viral load	CD4	CrAg†	Xpert MTB/RIF	eGFR	ALT	FBC	HbSAg
1	Eastern Cape	EC1	No	No	No	Yes	Yes	Yes	Yes	No
2	**-**	EC2	No	No	No	Yes	Yes	Yes	Yes	No
3	**-**	EC3	No	No	No	Yes	Yes	Yes	Yes	No
4	Limpopo	LP1	No	No	No	Yes	Yes	Yes	Yes	No
5	**-**	LP2	No	No	No	Yes	Yes	Yes	Yes	No
6	Free State	FS1	No	No	No	Yes	Yes	Yes	Yes	No
7	**-**	FS2	No	Yes	Yes	Yes	Yes	Yes	Yes	No
8	North West	NW1	No	Yes	Yes	Yes	Yes	Yes	Yes	No
9	**-**	NW2	No	No	No	Yes	Yes	Yes	Yes	No
10	Northern Cape	NC1	No	Yes	Yes	Yes	Yes	Yes	Yes	No
11	**-**	NC2	No	No	No	Yes	Yes	Yes	Yes	No
12	**-**	NC3	No	No	No	Yes	Yes	Yes	Yes	No
13	**-**	NC4	No	Yes	Yes	Yes	Yes	Yes	Yes	No
14	Western Cape	WC1	No	No	No	Yes	Yes	Yes	Yes	No
15	**-**	WC2	No	Yes	Yes	Yes	Yes	Yes	Yes	No
16	**-**	WC3	No	No	No	Yes	Yes	Yes	Yes	No
17	**-**	WC4	No	No	No	Yes	Yes	Yes	Yes	No
18	**-**	WC5	No	No	No	Yes	Yes	Yes	Yes	No
19	**-**	WC6	No	No	No	Yes	Yes	Yes	Yes	No

† Reflexed CrAg screening offered routinely as from 01 July 2017.

CD4, Cluster differentiation 4; CrAg, Crytococcal antigen; Xpert MTB/RIF, GeneXpert ; eGFR, Estimated Glomerular Filtration Rate; ALT, Alanine Transminase; FBC, Full Blood Count; HbSAg, Hepatitis B Surface Antigen.

### Antiretroviral therapy-related testing currently provided by candidate laboratories

All candidate laboratories (*n* = 19) provide Xpert MTB/RIF, creatinine (including a calculated estimated glomerular filtration rate), ALT and FBC testing at present (Table 1). None of the candidate laboratories, however, offer HIV viral load or Hepatitis B surface antigen testing. Five candidate laboratories already offer CD4 testing, namely FS2, NW1, NC1, NC4 and WC2.

### Candidate laboratories in close proximity

**FIGURE 4:**
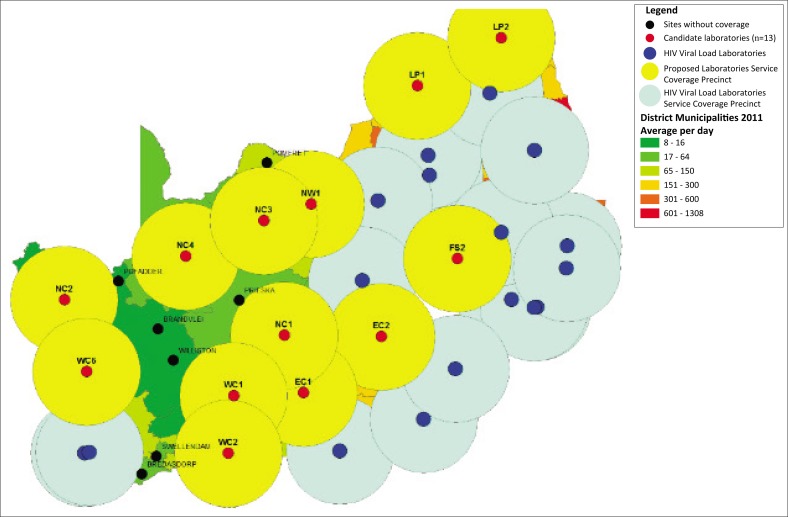
Analysis of additional coverage provided by the 13 candidate laboratories that would provide the required diagnostic support for antiretroviral therapy services, South Africa.

Four clusters were identified following visual inspection. Inter-laboratory distances ranged from 52.6 to 83.1 kilometres. Similarly, travel times ranged from 44 to 62 min (Table 2). Consolidation of testing was proposed at the NW1, WC2 and FS2 laboratories as they currently offer CD4 testing. The EC2 laboratory performed 28 000 Xpert MTB/RIF, creatinine, ALT and FBC tests, compared to 10 000 for the EC3 laboratory in FY2015/16. Therefore, the recommendation for testing consolidation at the EC2 laboratory for this cluster. As such, the list of identified candidate laboratories (*n* = 19) was reduced to 13, with six laboratories removed due to close proximity to the proposed site, namely NW2, WC3, WC4, WC5, FS1 and EC3. The remaining 13 laboratories were referred to as revised candidate laboratories (Figure 4).

**TABLE 2:** Google Maps distances and travel times between candidate laboratories in close proximity across the four clusters identified, South Africa.

Candidate consolidated laboratory to improve coverage	Candidate laboratories in close proximity	Distance	Travel time
NW1	NW2	74.2 km	51 min
WC2	WC3	62.1 km	56 min
**-**	WC4	52.6 km	44 min
**-**	WC5	59.4 km	48 min
FS2	FS1	80.0 km	62 min
EC2	EC3	83.1 km	53 min

NW, North West ; WC, Western Cape; FS, Free State; EC, Eastern Cape; km, kilometres; min; minutes.

### Analysis of test volumes and capacity required for CD4 and HIV viral load testing for the revised candidate laboratories

Daily HIV viral load referrals for the revised candidate laboratories (*n* = 13) ranged from 5 (NC2) to 205 (FS2) samples per day (Table 3). The FS2, NW1 and WC2 laboratories reported daily volumes of 205 (FS2), 138 (NW1) and 80 (WC2). The NC2, WC1 and WC6 laboratories all reported lower daily volumes, ranging from five to seven samples per day.

**TABLE 3:** Daily HIV viral load and CD4 test volumes, capacity required and testing tier for candidate laboratories, South Africa.

Number	Laboratory	HIV viral load			CD4		
	
		Daily volumes	Proposed platform	Quantity	Daily volumes	Proposed platform	Quantity
1	EC2	36	GX16	1	42	BC Aquios	1
2	LP1	26	GX16	1	25	BC Aquios	1
3	LP2	23	GX16	1	18	BC Aquios	1
4	FS2	205	GX16	4	83	BC Aquios	1
5	NW1	138	GX16	3	54	BC Aquios	1
6	NC1	18	GX16	1	18	BC Aquios	1
7	NC2	5	GX4	1	6	BD FacsPresto	1
8	NC3	46	GX16	1	56	BC Aquios	1
9	NC4	25	GX16	1	19	BC Aquios	1
10	WC1	7	GX4	1	8	BD FacsPresto	1
11	EC1	10	GX4	1	8	BD FacsPresto	1
12	WC6	6	GX4	1	9	BD FacsPresto	1
13	WC2	80	GX16	2	85	BC Aquios	1

LP, Limpopo; NW, North West; WC, Western Cape; FS, Free State; EC, Eastern Cape; GX, GeneXpert; BD, Beckton Dickinson; BC, Beckman Coulter

For CD4 testing, four laboratories with lower daily volumes (6–9 samples) would cope with the low throughput of a BD FacsPresto platform. The remaining laboratories (*n* = 9) would have sufficient capacity with the BC Aquios platform to perform up to 120 samples per day (daily volumes ranged between 18 and 85).

### Identify remaining antiretroviral therapy-related testing coverage gaps following the introduction of the antiretroviral therapy-related test repertoire at the revised candidate laboratories

The majority of the ART-related diagnostic coverage gaps would be addressed with the exception of some rural municipal areas that include Brandvlei, Bredasdorp, Pofadder, Pomfret, Prieska, Swellendam and Williston, with daily HIV viral load test volumes of ≤ 6 samples per day (Figure 4).

## Discussion

This article describes an approach to establishing where service deficiencies lie across a national programme, using a step-by-step approach. Each step provides detail of the analysis undertaken to make the final decision to identify and address coverage gaps. The work is by no means fully comprehensive but should be viewed as a guideline of how to approach the mammoth task of where to start rolling out laboratory services across a national programme, irrespective of whether the programme is a vast network of laboratories that may be required for extensive ART support services or a smaller network that involves other clinical laboratory testing.

The adequacy of ART-related diagnostic coverage is best addressed across a defined test repertoire that provides access to all testing required for ART initiation and monitoring of HIV-positive patients enrolled on ART. Addressing the coverage gaps for a single test would not have the desired impact on ART initiation, as patients would be required to wait for those tests results that are not included in the local test repertoire.

The 250 km service coverage precinct was based on the hub-and-spoke logistics model used within the NHLS.^42^ On average, samples are in transit with the courier for up to 3.5 hours before reaching the local NHLS laboratory. To meet the minimum six-hour requirement for plasma HIV viral load samples, a service coverage precinct of 250 km was allocated (~2.5 h travel time).

An advantage of providing ART-related coverage is the ability to support integrated patient care envisaged by the Ideal Clinic Initiative.^43^ The Ideal Clinic Initiative is a National Department of Health programme that aims to systematically improve and correct deficiencies in primary health care facilities in the public sector as a mechanism to promote health and to prevent illnesses and further complications through health promotion, early detection, treatment and appropriate referral.^43^ An Ideal Clinic is an integrated primary health care facility with good infrastructure, adequate staff, adequate medicine and supplies, good administrative processes, and sufficient adequate bulk supplies.^43^ The success of the planned National Health Insurance will depend on a well-functioning primary health care system.^43^ An ideal clinic should use clinical policies, protocols and guidelines to deliver integrated health care services. This includes the Integrated Clinical Services Management approach that builds on the strengths of the HIV programme to deliver integrated care to patients by taking a patient-centric view.^43^ Integrated care removes the requirement for patients to present for care on multiple visits (e.g. well baby clinic [immunisation], prevention of mother-to-child transmission of HIV check-ups and treatment for non-communicable diseases), rather allowing for a patient with multiple linked conditions to be treated in a single visit.^44^

Integrated care could be supported by providing decentralised ART-related diagnostics. Improving the capacity of local haematology and chemical pathology testing would have a broader impact for the diagnosis of chronic diseases such as diabetes and cardiovascular diseases.^44^ Many of the routine platforms used by the laboratories could easily be extended to include additional tests such as fasting glucose and troponin-T. Additionally, some of the polyvalent platforms could be used for multiple tests. For example, the Xpert platform is suitable for HIV viral load testing at the candidate laboratories due to the lower daily volumes. As laboratory personnel have already been exposed to this platform for tuberculosis testing, they have acquired the skills to competently use this platform, so that adding the additional test should be relatively easy to implement. Should laboratory test volumes increase dramatically in future, either additional Xpert platforms could be used or the laboratory could migrate to a higher-throughput Roche or Abbott HIV viral load platform.^27,45^ For CD4 testing, the Aquios and BD FacsPresto platforms^24,46^ are proposed, given the current daily volumes. Both platforms are relatively easy to use and have been tested at remote laboratories.^46^ For Hepatitis B surface antigen testing, the Vikia lateral flow test (bioMerieux, Marcy l’Etoile, France) is proposed as an option. In South Africa, CrAg screening using the Lateral Flow Assay (IMMY, Norman, Oklahoma, United States) is currently offered by all CD4 laboratories as a reflex test following a CD4 ≤100 cells/µL. Irrespective of the CD4 technology used, the simplicity of the CrAg lateral flow test will facilitate easy implementation into a candidate laboratory as the test was primarily designed for use at the POC.

For additional ART monitoring tests, such as fasting cholesterol and triglycerides, it would be beneficial to perform these tests at larger referral NHLS laboratories, where both the required platforms and mono-specialist trained medical technologists are available. These test results are not required for initiation onto ART, but can be available for the subsequent clinic visit. If a decision is made to implement fasting cholesterol and triglyceride testing in a candidate laboratory, the Alere Cholestech LDX analyser (Alameda, California, United States) has been evaluated and found to provide clinically-equivalent results when compared to conventional laboratory based platforms.^47^ However, this platform does not provide a triglycerides result. Suitable low throughput platforms for triglyceride testing are not currently available, affirming the need to refer these tests to a centralised facility.

Outcomes from the CD4 ITSDM suggest that using existing NHLS laboratories first to extend diagnostic coverage before investigating POC hubs or decentralised POC (true-POC) could reduce costs related to fit-for-implementation site development, training and competency costs and improve capacity.^31,48^ This approach is similar to public health initiatives such as extending HIV counselling and testing to the Clicks® chain of stores in South Africa to improve access to HIV counselling and testing services.^49^ Additionally, the ITSDM balances high-throughput testing in urban areas with decentralisation in hard-to-reach rural areas. This study has demonstrated that the ITSDM could be used to both identify and address coverage gaps for national programs other than CD4, such as HIV viral load, tuberculosis and non-communicable diseases.

Once approval has been obtained to implement ART-related testing at the proposed revised candidate laboratories, extensive planning is required, including site visits, gap analysis and identifying and addressing staffing requirements. In the implementation phase, activities include laboratory preparation, staff recruitment, analyser procurement followed with delivery and set-up, user training and finally, competency assessment and verification (fit-for-purpose). These activities need to be synchronised through a harmonising coordinating body, described as Tier-6 in the ITSDM, which will coordinate, streamline and standardise all processes relating to implementation, training and quality. In the South African context, this harmonising tier is provided for by the National Priority Programme Unit of the NHLS. Additional cadres of staff, such as the phlebotomist technicians or medical technicians,^50^ could be considered to extend testing by recruiting and training community members for long-term sustainability where POC facilities may be needed to extend laboratory services in hard-to-reach areas.^51^

Improved access to ART-related testing should improve the overall access to laboratory service delivery to support clinical services in these remote areas. Furthermore, building capacity for ART-related testing could be an important stepping stone to improving access for the diagnosis of chronic diseases such as cardiovascular diseases, diabetes, chronic respiratory conditions and cancer.^52^

The algorithm is intended to be an iterative process. Once a solution has been implemented, the algorithm could be repeated until all coverage gaps are addressed. If the step-by-step methodology is automated on the corporate data warehouse, this approach could be applied to multiple tests in real time, enabling key coverage decisions within the NHLS.

### Limitations

This study was a desktop exercise and is based only on data extracted for FY2015/16. It does not reflect NHLS plans for expansion of services but is merely an exercise to demonstrate the processes followed in the development of the ITSDM and how the model can be applied to inform on other laboratory services.

The concepts and methodology described here can be developed in any organisation to provide real time analysis of coverage gaps in a national network of laboratories as well as extend the concept to other laboratory test services with minimal effort. The visual assessment of candidate laboratories in proximity could be further replaced by an automated proximity analysis, as described elsewhere.^36,37,53^ Finally, the model outcomes presented here need vigorous review and testing by senior area managers using their local insights to identify constraints such as space availability, logistics routing and staffing availability to assess the viability of each proposed testing site.

Costing of the various tiers, including decentralised ITSDM, has been undertaken and published elsewhere^31,50^ with evidence of improved service delivery provided by extending and decentralising testing.^48^ Further health economic studies would be of value to determine both the implementation and incremental cost of providing decentralised ART-related testing as described in this work. Both a bottom-up and top-down costing analysis would be required to assess costs using a provider perspective, especially in the context of recent local data which suggests a large advanced HIV disease burden in South Africa,^54^ with the ambitious UNAIDS 90-90-90 HIV treatment goals proposed for the third world.^55^

### Conclusion

This article has demonstrated that a service coverage precinct approach could be used to identify coverage gaps for an ART-defined test or -relevant repertoire depending on the programmatic requirements that need to be addressed. The ITSDM approach could be used as a starting point to define best practices to introduce sustainable testing as part of a national service extending laboratory coverage to other chronic disease testing. This approach addresses coverage gaps in a cost effective manner.
